# Editorial: Neuroscience-inspired visual sensing and understanding

**DOI:** 10.3389/fnins.2023.1270990

**Published:** 2023-08-18

**Authors:** Qingbo Wu, King Ngi Ngan, Weisi Lin, Lei Bai

**Affiliations:** ^1^School of Information and Communication Engineering, University of Electronic Science and Technology of China, Chengdu, China; ^2^School of Computer Science and Engineering, Nanyang Technological University, Singapore, Singapore; ^3^AI for Science Department, Shanghai AI Laboratory, Shanghai, China

**Keywords:** visual sensing, human vision system, visual perception and cognition, visual attention model, neural network

Visual sensing and understanding play a fundamental role in building an intelligent vision system. Despite great progress in recent years, it is still extremely challenging for existing machine agents to perceive and describe visual information quickly and accurately in the wild open world, in comparison with their biological counterparts, i.e., the human vision system (HVS). When confronted with complex scene changes and diverse visual content, the HVS can easily evaluate the visual inputs visibility, dynamically adapt the retina for higher perceptual quality, quickly locate all interesting objects, and accurately parse their semantic relationships. These abilities attract us to make use of the neural mechanisms of the HVS.

Substantial research effort has been devoted to visual sensing and understanding in the related scientific communities. The most recent success has benefited from a deep neural network to mimic the simplified architecture of HVS but works like a “black box” mapping model. Existing methods usually suffer from significant performance degradation. The recent development in neuroscience has enriched our knowledge about the HVS and offered great opportunities in building interpretable and trustworthy intelligent vision systems; especially for embedding the neural mechanism of the HVS into visual perception modeling, imaging quality enhancement, neural network design, and so on. These have become the frontiers in intelligent vision research.

This Research Topic is a collection of articles concerning neuroscience-inspired visual sensing and understanding, which aim to develop interpretable and trustworthy intelligent vision systems. The finally accepted articles conduct this exploration from three aspects: 1. Neuroscience studies of visual perception and recognition mechanism; 2. Neuroscience-inspired visual attention models and applications; 3. Neuroscience-inspired neural network architecture and learning technologies. In the following, we present a brief overview and discussion of the accepted articles.

## Neuroscience studies of visual perception and recognition mechanism

In recent years, visual perception and recognition mechanisms have received increasing attention in improving the interpretability and credibility of machine vision algorithms. In this process, the latest neuroscience studies are crucial in offering us explicit computational models, which are capable of describing various complex behaviors of the HVS.

Firstly, Gundavarapu and Chakravarthy focused on the motion sensing of medial superior temporal neurons, and studied the selective cortical responses of the primate motion pathway. A hierarchical neural field framework with three models is developed to better recognize the type of optic flow. Then, Wang et al. investigated the HVS's characteristics of temporal dynamics in browsing omnidirectional images, and proposed to capture dynamically attentive regions based on the gravity law, which predicts visual scan path. Moreover, they developed an objective no-reference quality assessment model to evaluate the viewing experience of omnidirectional images through head-mounted displays.

## Neuroscience-inspired visual attention models and applications

The attention mechanism has been an active research field for many years, which contributes to highly accurate and efficient visual signal processing and understanding. Recent studies also present encouraging progress in neuroscience-inspired visual attention models and applications.

Zhang et al. focused on the edge detection task and introduced a modulation coding network inspired by the attention mechanism of the ventral pathway, which selectively extracts edge-aware features under the guidance of global image information. Meanwhile, a dual decoding network is proposed to integrate both the top-down and bottom-up features to simulate the functions of the inferior temporal cortex. Zhou et al. focused on medical image segmentation and proposed an attention-based residual depth-wise separable convolution, which applies the attention gate to locate the most significant features and eliminate redundancy. Both the segmentation performance and computational efficiency were improved with this model.

## Neuroscience-inspired neural network architecture and learning technologies

More and more neuroscience-inspired neural network architectures and learning technologies are emerging, and this deepens our understanding of deep learning. These new explorations play an important role in the robustness and reliability of intelligent vision systems.

Pi et al. explored the vulnerability of existing deep neural networks and proposed a relational graphs ensemble adversarial attack method to improve the adversarial transferability across multiple models. This method employed an ensemble learning strategy and derived the optimal attack direction from the linear combination of multiple models, whose weights are determined by the dependency of each pair of models. Gai et al. introduced a global-local representation learning network, which is composed of the convolution and transformer layers. On the one hand, the multi-scale convolution and pooling enrich the local geometric detail of shallow features. On the other hand, the multi-head self-attention based transformer layers better encode the global semantic information. Experiments on multiple medical image segmentation databases verify the effectiveness of this neural network architecture.

We hope that readers find this Research Topic timely, informative, and enlightening in exploring neuroscience-inspired visual sensing and understanding, and it would attract more interest and trigger more investigation in the related areas.

## Author contributions

QW: Writing—original draft. KN: Writing—review and editing. WL: Writing—review and editing. LB: Writing—review and editing.

